# Covalent Inhibitors from Saudi Medicinal Plants Target RNA-Dependent RNA Polymerase (RdRp) of SARS-CoV-2

**DOI:** 10.3390/v15112175

**Published:** 2023-10-30

**Authors:** Ahmed H. Bakheit, Quaiser Saquib, Sarfaraz Ahmed, Sabiha M. Ansari, Abdullah M. Al-Salem, Abdulaziz A. Al-Khedhairy

**Affiliations:** 1 Department of Pharmaceutical Chemistry, College of Pharmacy, King Saud University, P.O. Box 2457, Riyadh 11451, Saudi Arabia; abakheit@ksu.edu.sa; 2 Zoology Department, College of Sciences, King Saud University, P.O. Box 2455, Riyadh 11451, Saudi Arabia; alsalem1985@hotmail.com (A.M.A.-S.); kedhairy@ksu.edu.sa (A.A.A.-K.); 3 Department of Pharmacognosy, College of Pharmacy, King Saud University, P.O. Box 2457, Riyadh 11451, Saudi Arabia; ahmsarfaraz@gmail.com; 4 Botany & Microbiology Department, College of Sciences, King Saud University, P.O. Box 2457, Riyadh 11451, Saudi Arabia; sabihamahmood003@gmail.com

**Keywords:** SARS-CoV-2, COVID-19, medicinal plants, RdRp, docking, MD simulation, phytochemicals

## Abstract

COVID-19, a disease caused by SARS-CoV-2, has caused a huge loss of human life, and the number of deaths is still continuing. Despite the lack of repurposed drugs and vaccines, the search for potential small molecules to inhibit SARS-CoV-2 is in demand. Hence, we relied on the drug-like characters of ten phytochemicals (compounds **1**–**10**) that were previously isolated and purified by our research team from Saudi medicinal plants. We computationally evaluated the inhibition of RNA-dependent RNA polymerase (RdRp) by compounds **1**–**10**. Non-covalent (reversible) docking of compounds **1**–**10** with RdRp led to the formation of a hydrogen bond with template primer nucleotides (A and U) and key amino acid residues (ASP623, LYS545, ARG555, ASN691, SER682, and ARG553) in its active pocket. Covalent (irreversible) docking revealed that compounds **7**, **8**, and **9** exhibited their irreversible nature of binding with CYS813, a crucial amino acid in the palm domain of RdRP. Molecular dynamic (MD) simulation analysis by RMSD, RMSF, and Rg parameters affirmed that RdRP complexes with compounds **7**, **8**, and **9** were stable and showed less deviation. Our data provide novel information on compounds **7**, **8**, and **9** that demonstrated their non-nucleoside and irreversible interaction capabilities to inhibit RdRp and shed new scaffolds as antivirals against SARS-CoV-2.

## 1. Introduction

SARS-CoV-2, also called the 2019 novel coronavirus (2019-nCoV), responsible for causing COVID-19 disease, was first detected in late December 2019 in Wuhan (Hubei, China), a city with a large market for exotic meats from live animals as well as natural products from plants widely used in local food production [1]. Although the source of SARS-CoV-2 is still unknown, its genome sequence has revealed its closest similarity (96.2%) with the bat SARS-like coronavirus [2,3,4]. Considering the massive increase in COVID-19 cases globally, the WHO has declared a pandemic outbreak in 2020. As of 18 October 2023, 6,972,152 humans have lost their lives due to SARS-CoV-2 [5]. Some infected patients showed the development of acute respiratory distress syndrome (ARDS), which causes pneumonia, septic shock, and death [6]. In addition, ARDS also triggers a cytokine storm in the infected patients that necessitates intensive care [7]. SARS-CoV-2 utilizes two major pathways to gain entry into host cells. Firstly, it has the ability to fuse with the plasma membrane to enter the cells. On the other hand, the second pathway is by fusion with the endosomal membrane, which depends on the proteases in its local environment, indicating the flexibility of spike proteins response to the varying signal proteins [8,9]. Mechanistically, SARS-CoV-2 entry in the host cells relies on the binding of the transmembrane spike (S) glycoprotein (forms homotrimers) [10] to a specific cellular receptor, as well as subsequent S protein priming by cellular proteases. To do so, SARS-CoV-2 recruits ACE2 as a receptor for cellular entry. It has also been found that S protein and ACE2 receptor binding affinity is correlated with viral replication rate and disease severity [2,9,11]. Consequently, ACE2 is a potential target for the entry of SARS-CoV-2 in the host cell.

SARS-CoV-2 falls under the β genus of the coronavidae family and contains ≈30 kb single-stranded RNA (+ve sense) as its genetic material [12]. SARS-CoV-2, all together, contains 14 open reading frames encoding 27 varying types of proteins [13,14]. SARS-CoV-2 transcribes its genome to produce ≈800 kDa of large polyproteins that undergo proteolytic cleavage to make several non-structural proteins (NSPs) crucial for its replication [15,16]. Primarily, proteolytic enzymes like main protease (M^Pro^) and papain-like protease (PL^Pro^) cleave the long polypeptide to release sixteen (16) NSPs [17]. Among the 16NSPs, NSP12 or RNA-dependent RNA polymerase (RdRp) is complexed with NSP7/8 to facilitate viral replication [18]. It is intriguing that despite the high frequency of mutation in SARS-CoV-2, RdRp sequences are highly conserved due to the reality that changes in such an essential protein are deadly for the virus [17,19,20]. Also, viral RdRp lacks a human homologue [21]. Consequently, RdRp has been regarded as one of the prospective targets for drug development against SARS-CoV-2, although it is also regarded as a prospective target for drugs either approved or under clinical trial against influenza, hemorrhagic fever, hepatitis C, and respiratory syncytial-causing viruses [22,23,24,25,26,27,28,29].

With the availability of genetic and structural information on SARS-CoV-2, scientists across the globe have rushed towards the development of drugs as antiviral inhibitors [2]. In particular, computational approaches, including the docking methods, have offered substantial benefits in defining repurposed drugs against SARS-CoV-2. However, the advantage of docking or virtual screening of drugs without experimental validation underscore the high-risk prediction that carries a greater rate of false-positive outcomes [30]. One of the main shortcomings of universal energy-based scoring functions in docking is their inability to fully account for the protein conformational changes that occur upon binding with a ligand. Nonetheless, the search for natural drugs for their effectiveness against SARS-CoV-2 has also started [13,31]. For ages, herbal medicines and purified phytochemicals have been implicated in the discovery of novel drugs and efficient drugs based on the structure of natural compounds [32]. In this connection, there are several natural compounds that have exhibited efficacy in inhibiting SARS-CoV entry into host cells. Natural sources containing an anthraquinone compound and emodin have been shown to inhibit SARS-CoV binding with the receptor ACE2 [33]. In line with this concept, 57 phytochemicals from the public database showed their binding with RdRp, 3CL^Pro^, ACE2, and the spike glycoprotein of SARS-CoV-2. The analysis suggested that apigenin-o-7-glucuronide and ellagic acid from *Eucalyptus globulus*, eudesmol and viridiflorene from *Vitex negundo*, and vasicolinone and anisotine from *Justicia adhatoda* were the potent ligands that bind strongly to the target proteins of SARS-CoV-2 [34]. Docking analysis of *Eucalyptus* and *Corymbia* species essential oils has exhibited potent binding with 3CL^Pro^ [35]. SARS-CoV-2 NSP exhibited the lowest free binding energies with nine phytochemicals from *Withania somnifera*, which indicated them as prospective inhibitors for the virus [36]. Four phytochemicals from Chinese herbs (glycyroside, licorice glycoside E, (-)-medicocarpin, and diisooctyl phthalate) bonded strongly with RdRp [18]. Virtual screening of phytochemicals (pedaliti, quercetin, gallic acid) binds with several amino acids in the active pocket of RdRp [37]. Rutin, a flavonoid, has shown stronger binding affinity towards different amino acid residues of RdRp via the formation of a hydrogen bond and van der Waals force [38]. In silico study has revealed that a green tea polyphenol, epicatechin gallate, interacted with RdRP through hydrogen bond and hydrophobic interactions [39].

Despite the availability of a few vaccines and repurposed drugs, the search for effective drugs against COVID-19 is still in progress. Rather than performing virtual screening of phytochemicals from a plethora of databases, we exclusively selected ten (compounds **1**–**10**) phytochemicals that we had previously isolated and purified from Saudi medicinal plants and evaluated their pharmacological effects in different test models, including their anti-viral properties against the hepatitis B virus [40,41,42]. Hence, in the current study, compounds **1**–**10** were assessed as small molecules inhibitors targeting RdRp via non-covalent and covalent docking protocols, quantifying the stability of complexes by molecular dynamic (MD) simulations by measuring RMSD, RMSF, and Rg.

## 2. Materials and Methods

### 2.1. Details of Phytochemcials and Prepartion of Ligands

In total, we selected ten compounds (**1**–**10**) from Saudi medicinal plants that were isolated and purified earlier by our lab [40,41,42]. Table 1 lists the details of compounds **1**–**10**. For the computational studies, MarvinSketch (version 21.19.0) was used for drawing the 2D structures of each compound [43]. Furthermore, the 3D structure of all compounds was developed through the Molecular Operating Environment (MOE)-builder tool, a part of the MOE suit (version 2015.10) (Chemical Computing Group Inc., Montreal, QC, Canada). Energy minimization of all compounds was performed by the use of MMFF994x with a root mean square (RMS) of <0.05 kcal/mol Å^−1^. In addition, PM3 and AM1 processes were implemented for calculating the partial charges, as well as atomic charges (partial) for ligands and atoms.

### 2.2. Preparation of RdRP

The RNA-dependent RNA polymerase (RdRp) (PDB ID: 7BV2) crystal structure was downloaded from the Protein Data Bank [44]. The resolution of RdRp was 2.50 Å, and remdesivir (RTP) was a co-crystalized ligand in this protein. The crystal structure was implemented for the removal of water molecules. Subsequently, it was applied for protein correction through the structure preparation module, and lastly 3D protonated. Identification of the active site in RdRP was achieved via its co-crystalized ligand. Prior to the commencement of the initial docking protocol, the docking protocol was employed to re-dock RdRp co-crystalized with its ligand in the binding site. Acceptance of the docking protocol was performed by observing the <3 value of the root-mean-square deviation (RMSD) for both co-crystalized ligands and the redocked ligands through the MOE scientific vector language script [45]. 

### 2.3. Docking Experiments

#### 2.3.1. Docking Validation

RdRP provided a foundation to identify small molecules from the cohort of compounds **1**–**10**. We implemented MOE software (version 2015.10) to validate docking protocol. RTP, as a ligand, was re-docked through the docking protocol to affirm its alignment and confirmation of an active site in RdRp [46]. Factors like triangle matcher, GBVI/WSA dG, ligand atoms, and London dG were chosen for the fields of site, score, and method (placement). The rest of the other parameters were kept to their default values. Within each cycle of the triangle matcher, the ligand exhibiting the best conformation and apt orientation was selected. 

#### 2.3.2. Non-Covalent Docking

MOE software suite (version 2015.10) was used for performing the irreversible (non-covalent) docking of RdRp with compounds **1**–**10**. Preparation of RdRp was initiated first in non-covalent docking. RdRP was then protonated through the Protonate 3D method [47]. Afterwards, RdRp was partially charged by the use of the AMBER10:EHT force field. Later, RdRP and compounds **1**–**10** were subjected to non-covalent docking via the implementation of the same protocol used in the validation of the docking method.

#### 2.3.3. Irreversible (Covalent) Docking 

Covalent docking of RdRp and compounds **1**–**10** was accomplished with the covalent module in the MOE software suite (version 2015.10). Firstly, RdRp was prepared and protonated, with the subsequent addition of partial charges to it using the AMBER10:EHT force field. Precisely, in the covalent docking protocol, reactive sites were selected that affirmed that pyrophosphate (POP) and two magnesium (Mg^++^) ions were the reactive sites. POP was selected by focusing on one of the oxygen atoms, which showed orientation in the hydrophobic pocket. Subsequently, the covalent docking protocol was run by implementing the Michael addition reaction. The best conformation of ligands falling under the criteria to form covalent bonds with atoms in the reactive sites, along with high scores, were retained for MD simulation studies [48,49,50,51].

### 2.4. MD Simulation Studies

MD simulations analysis was performed by Nanoscale molecular dynamics (NAMD) software (version 3.0) [52]. MD simulation was performed for ligands (compounds **7**, **8**, and **9**) complexed with the target protein (RdRp), as well as RdRp alone. With the use of the MOE software suite (version 2015.10), MD simulations were commenced by minimizing the energy of compounds **7**, **8**, and **9** complexed individually with RdRp. Configuration files for complexes were generated by CHARMM-GUI [53]. Subsequently, the ligand–target complexes were subjected to parameterization through a CHARMM General Force Field (CGenFF) [54,55,56]. The all-atom additive CHARMM36 force field was used to generate the topology of RdRp complexed with compounds **7**, **8**, and **9** using [56]. Afterwards, all of the system was solvated by the TIP3P model [57]. The equilibration of the system energy (i.e., RdRp-ligands complexes, ions, solvent) was ensured, then subjected to minimization that was set at 10,000 iterations. The system was permitted to progress over 1,000,000 steps and 125,000 (run). The timeframe for equilibration was set for 125 ps through the NVT ensemble. NPT ensemble was used for producing MD simulations at a timescale of 50ns. Later, stability analysis of RdRp alone and complexed with the above ligands was performed, measuring the root-mean-square deviation (RMSD), root-mean-square fluctuation (RMSF), and radius of gyration (Rg).

## 3. Results

### 3.1. Details of Phytochemicals (Compounds ***1***–***10***)

**Figure 1:**
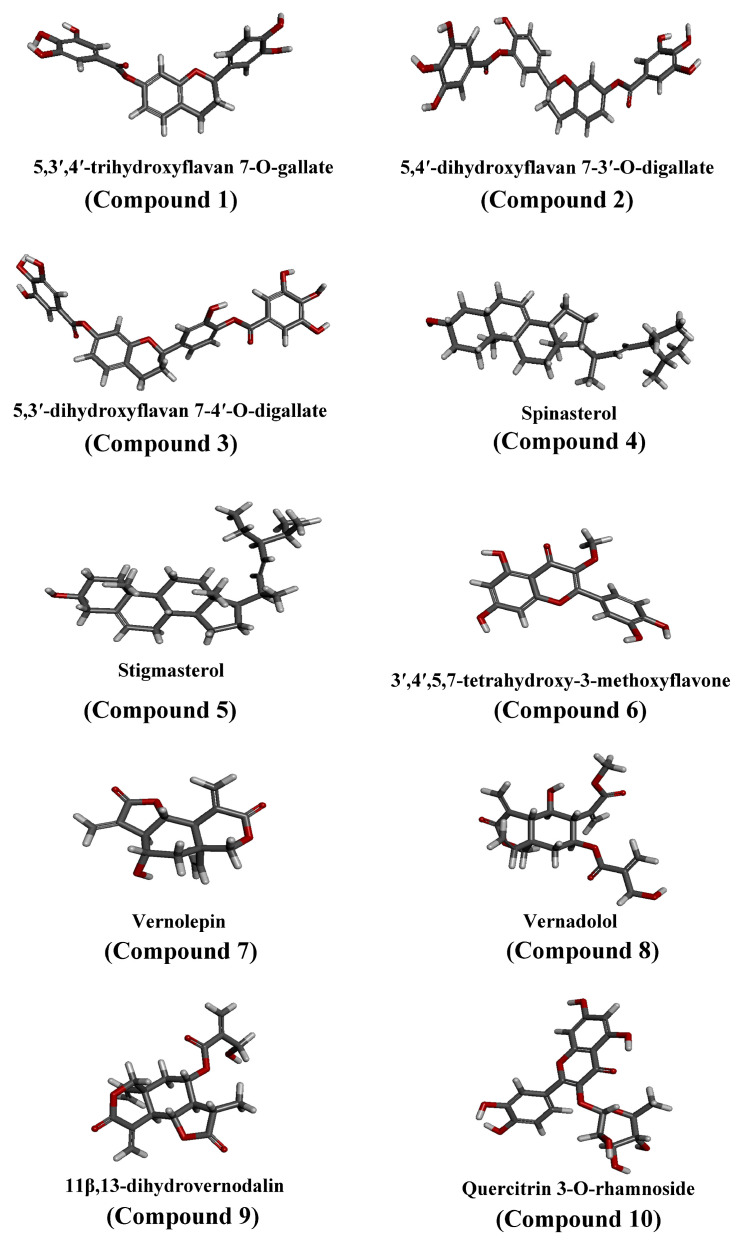
3D structure of phytochemicals (compounds **1**–**10**) from Saudi medicinal plants.

5,3′,4′-Trihydroxyflavan 7-O-gallate (compound **1**), 5,4′-dihydroxyflavan 7-3′-O-digallate (compound **2**), and 5,3′-dihydroxyflavan 7-4′-O-digallate (compound **3**) were isolated from Oncocalyx glabratus [40]. Spinasterol (compound **4**), stigmasterol (compound **5**), 3′,4′,5,7-tetrahydroxy-3-methoxyflavone (compound **6**), vernolepin (compound **7**), vernadolol (compound **8**), and 11β,13-dihydrovernodalin (compound **9**) were isolated from Baccharoides schimperi [41]. Quercitrin 3-O-rhamnoside (compound **10**) was isolated from Euphorbia schimperi [42]. The 3D structure of compounds **1**–**10** is shown in Figure 1. 

### 3.2. Active Site Explanation in RdRP

**Figure 2:**
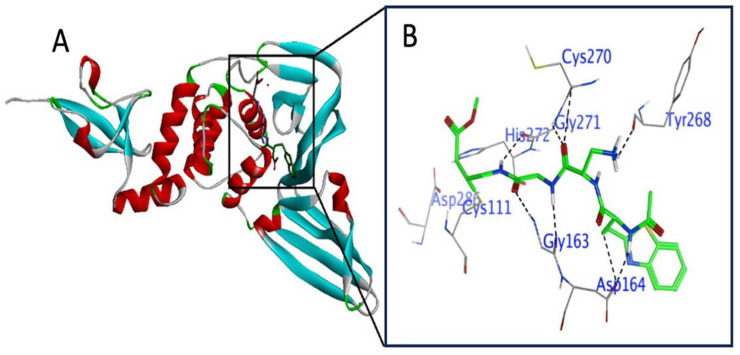
Active site in the SARS-CoV-2 target protein (RdRp). (**A**) Ribbon structure of RdRp (PDB ID: 7BV2) with ligand (remdesivir, RTP) indicating the active pocket in the target protein. (**B**) Magnified view of the active site of RdRp showing the catalytic dyad of residues interacting with RTP (green color). Black color (H-bond), dark red (H-π bond), dark blue (Van der Waals clashes), element color (atoms), residues are labeled as blue texts.

Before the start of docking studies with compounds **1**–**10**, re-confirmation of active sites in RdRp was performed. The active site of RdRp has been reported to be located in the palm subdomain containing conserved motifs (A, B, and C) [58,59]. As crucial residues, motif A contains ASP618; motif B contains ASP760, ASP761, and SER759; and motif C contains ASP812 [58,59]. In addition, Mg^++^ has also been reported to present as a catalytic metal ion in the active site [60] (Figure 2). 

### 3.3. Docking Protocol Validation with RdRP

Validation of the docking protocol was performed by redocking the co-crystal ligand remdesivir (RTP) with receptor protein (RdRp). RTP and RdRp binding redocking showed H–donor, H–acceptor, metal ion, ionic, and π–π stacking interactions. H-donor interaction developed at a distance of 2.98 Å between the N29 atom of the ligand and U10 in RdRp. At distances of 3.06, 2.91, and 34.4 Å, the N16, O19, and O32 atoms of the ligand showed H-acceptor interactions with U10, U10, and U20 in RdRp, respectively. Metallic and ionic interactions resulted at a distance of 1.93 Å between the O35 atom of the ligand and the MG1004 residue in RdRp. The five- and six-rings of the ligand showed π–π stacking with the U20 and A11 in RdRp (Table S1). Superimposition analysis showed that the co-crystalized ligand occupied the same position within the active site of crystalized remdesivir (RTP) in RdRp (Figure S1). The RMSD of the co-crystalized ligand superimposed with the crystalized ligand was found to be 0.970 Å for RdRp, which was less than <3 Å.

### 3.4. Rreversible (Non-Covalent) Docking of Compounds ***1***–***10*** with RdRP

**Figure 3:**
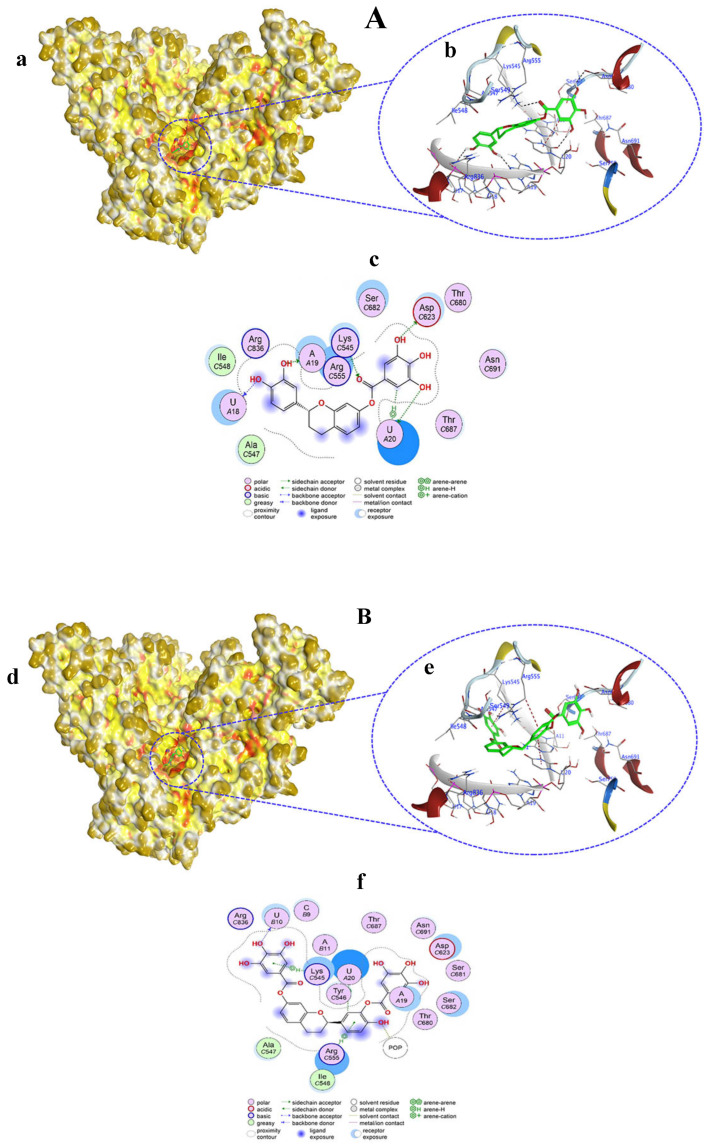
Surface representation showing the non-covalent docking of compound **1** (**A**(**a**)) and compound **2** (**B**(**d**)) with RdRp. Compounds **1** and **2** are green in color. Solvent-exposed regions of RdRp are dark yellow, hydrophobic regions are yellow, and polar regions are red in color. Magnified view showing the binding of compound **1** (**A**(**b**)) and compound **2** (**B**(**e**)) with the template primer nucleotides and residues in the active pocket of RdRp. Black color (H-bond), dark red (H-π bond), dark blue (Van der Waals clashes), element color (atoms), residues, and nucleotides are labeled as blue texts. Two-dimensional view of RdRp showing non-covalent binding with compound **1** (**A**(**c**)) and compound **2** (**B**(**f**)). Bonds and color descriptions are shown in the inset of the 2D image.

Compounds **1**–**10** were evaluated for their interaction properties with RdRp, in which non-covalent docking showed successful binding of the tested compounds. Compound **1** occupies the active site of RdRp and bound to the template primer nucleotides and residues as H–donor, H–acceptor, and H–π interactions (Figure 3A(a,b)). Compound **1** atoms O1, O5, and O37 bound to A19, U18, and U20 nucleotides at distances of 2.95, 2.93, and 3.09 Å via H–donor interaction. Also, the C39 atom of compound **1** bound with the U20 nucleotide by H–π interaction at a distance of 4.17 Å. Moreover, the O31 atom of compound **1** bound to the ASP623 residue by H–donor interaction at a distance of 2.93. On the other hand, the O41 atom bound to the LYS545 residue by H–acceptor interaction at a distance of 3.13 Å (Table S2, Figure 3A(c)). 

Compound **2** resided in the active site of RdRp and thereby exhibited an interaction with the template primer nucleotides as well as amino acid residues through H–donor, H–π, and π–H interactions (Figure 3B(d,e)). Compound **2** categorically bound to the U10 and U20 nucleotides of RdRp with its O13 and C62 atoms by H–donor and H–π interactions at distances of 2.87 and 4.48 Å, respectively. In addition, compound **2** also bound to POP1003, LYS545, and ARG555 amino acids of RdRp at distances of 2.78, 4.54, and 4.13 Å, respectively, via H–donor, π–H, and H–π interactions (Table S3, Figure 3B(f)). Compound **2** showed a docking score of −7.7369 kcal/mol, which was near the score of −8.870 kcal/mol obtained from the co-crystalized ligand remdesivir (RTP) with RdRp.

**Figure 4:**
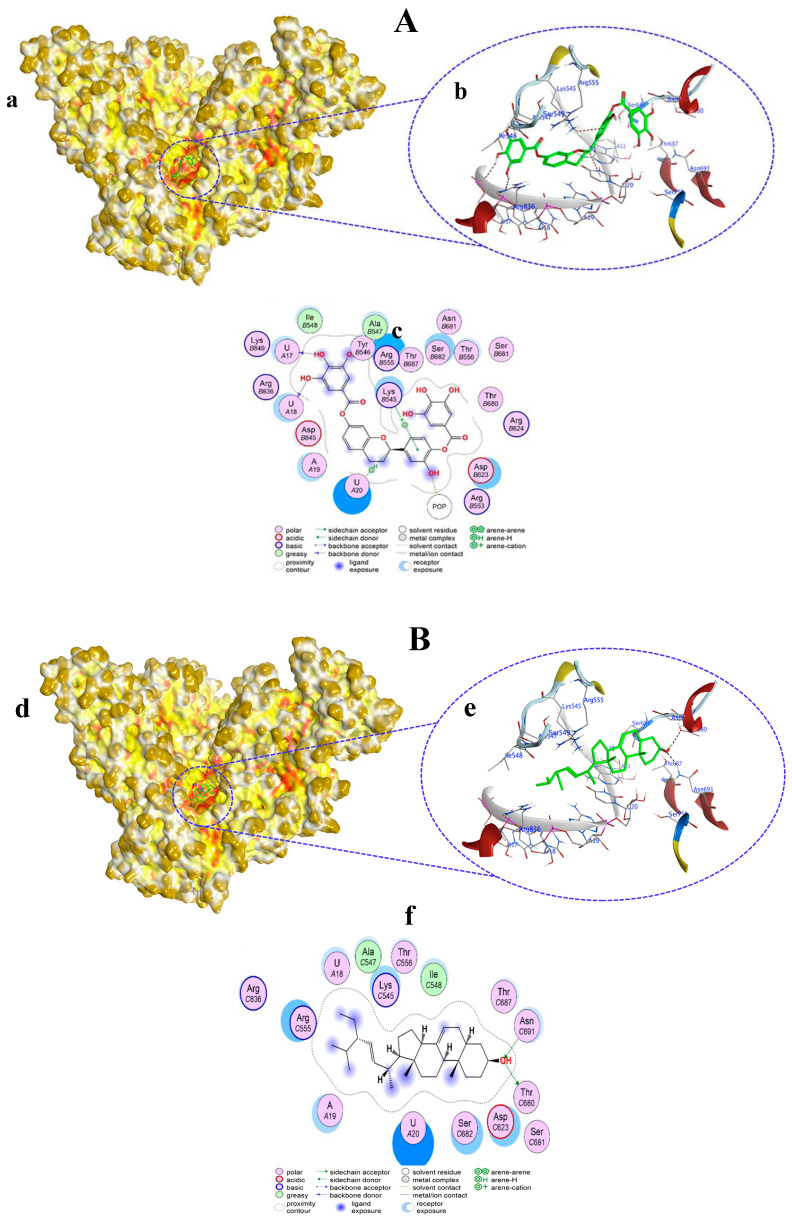
Surface representation showing the non-covalent docking of compound **3** (**A**(**a**)) and compound **4** (**B**(**d**)) with RdRp. Compounds **3** and **4** are in green color. Magnified view showing the binding of compound **3** (**A**(**b**)) and compound **4** (**B**(**e**)) with the template primer nucleotides and residues in the active pocket of RdRp. Two-dimensional view of RdRp showing non-covalent binding with compound **3** (**A**(**c**)) and compound **4** (**B**(**f**)).

Compound **3** bound to the amino acid residues as well as the template primer nucleotides of RdRp in the active site, chiefly by H–donor, H–π, and π–cation interactions (Figure 4A(a,b)). Compound **3** with its O10, O13, and C30 atoms bound to U18, U17, and U20 nucleotides through H–donor and H–π interactions at distances of 2.81, 3, and 4.34 Å, respectively. POP1003 and LYS545 residues in RdRp showed binding with O60 and the six-ring of compound **3** via H–donor and π–cation interactions at distances of 2.91 and 4.28 Å, respectively (Table S4, Figure 4A(c)). Compound **3** docking score was −8.0267 kcal/mol, which was nearly the same (−8.870 kcal/mol) shown by co-crystalized ligand remdesivir (RTP) with RdRp.

**Figure 5:**
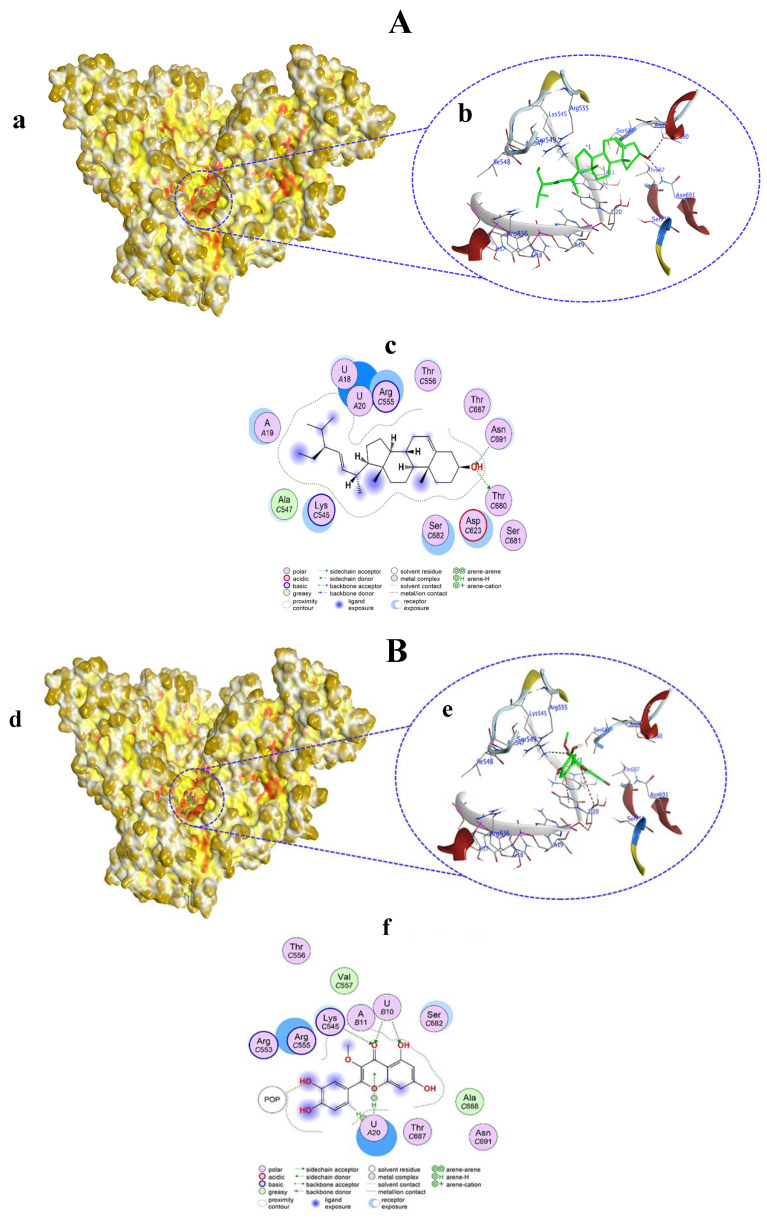
Surface representation showing the non-covalent docking of compound **5** (**A**(**a**)) and compound **6** (**B**(**d**)) with RdRp. Compounds **5** and **6** are in green color. Magnified view showing the binding of compound **5** (**A**(**b**)) and compound **6** (**B**(**e**)) with the template primer nucleotides and residues in the active pocket of RdRp. Two-dimensional view of RdRp showing non-covalent binding with compound **5** (**A**(**c**)) and compound **6** (**B**(**f**)).

Compound **4** (Figure 4B(d,e)) and compound **5** (Figure 5A(a,b)) both exhibited similar types of interactions as H-donors and H-acceptors within the active site of RdRp and bound only with the amino acids. H-donor compounds **4** (Table S5, Figure 4B(f)) and compound **5** (Table S6, Figure 5A(c)) established an interaction with the THR680 residue with its O1 atom at a distance of 3.1 Å. On the other hand, compounds **4** and **5** interacted with the ASN691 residue as H-acceptors at a distance of 2.94 Å. However, the docking scores of compounds **4** and **5** were different from each other (−5.0536 and −4.7243 kcal/mol, respectively).

Compound **6** docked in the active pocket of RdRp; thereby, it interacted with nucleotides and amino acid residues, acting as H–donor, H–acceptor, H–π, and π–H interactions (Figure 5B(d,e)). As H-acceptors, O1 and O27 atoms of compound **6** interacted with the U10 nucleotide at distances of 3.27 and 3.3 Å, respectively. Also, the O1 atom of compound **6** interacted with the LYS545 residue as an H-acceptor from a distance of 3.49 Å. C11 and the six ring of compound **6** showed H–π and π–H interactions with the U20 nucleotide. As an H-donor, only the O19 atom of compound **6** showed an interaction with the POP1003 residue in RdRp (Table S7, Figure 5B(f)). 

**Figure 6:**
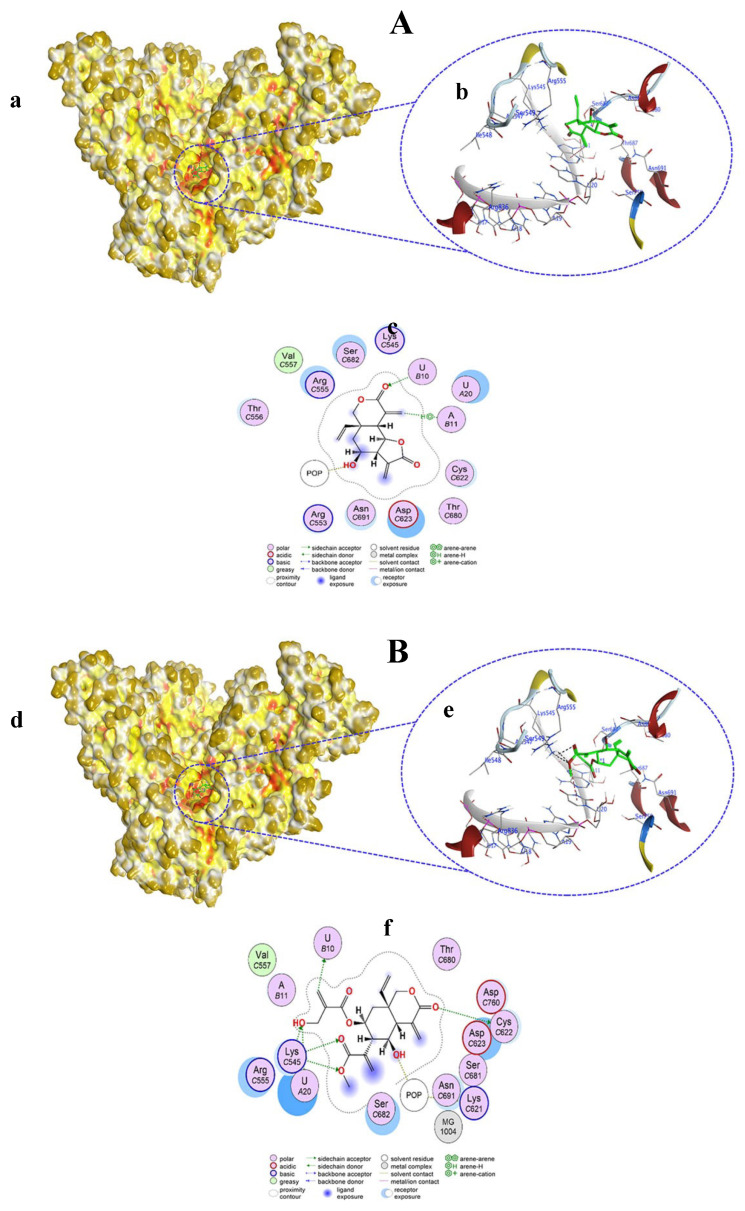
Surface representation showing the non-covalent docking of compound **7** (**A**(**a**)) and compound **8** (**B**(**d**)) with RdRp. Compounds **7** and **8** are in green color. Magnified view showing the binding of compound **7** (**A**(**b**)) and compound **8** (**B**(**e**)) with the template primer nucleotides and residues in the active pocket of RdRp. Two-dimensional view of RdRp showing non-covalent binding with compound **7** (**A**(**c**)) and compound **8** (**B**(**f**)).

Compound **7** fitted in the active pocket of RdRp and interacted with a few nucleotides and only one amino acid residue via H–donor, H–acceptor, and π–H interactions (Figure 6A(a,b)). Compound **7** O12 and C14 atoms bound to the U10 and A11 nucleotides of RdRp through H–acceptor and π–H interactions at distances of 2.99 and 4.88 Å, respectively. From a distance of 2.85 Å, the O32 atom of compound **7** acted as an H-donor to the POP1003 residue of RdRp (Table S8, Figure 6A(c)).

Compound **8** occupied the active pocket in the RdRp protein and interacted with only one nucleotide and several amino acid residues predominantly through H–donor and H–acceptor interactions as well as H–π interactions (Figure 6B(d,e)). As H-donors, O12 and O21 atoms of compound **8** interacted with CYS622 and POP1033 residues at distances of 3.55 and 2.86 Å, respectively. Conversely, the C42 atom of compound **8** interacted with the U10 nucleotide of RdRp at a distance of 3.43 Å. Acting as H-acceptors, O30 (3.2 Å), O31 (3.28 Å), and O48 (2.7 Å) atoms of compound **8** interacted with LYS545 amino acid residue. The O48 atom of compound **8** showed binding with the U10 nucleotide of RdRp by H–π interaction (Table S9, Figure 6B(f)). 

**Figure 7:**
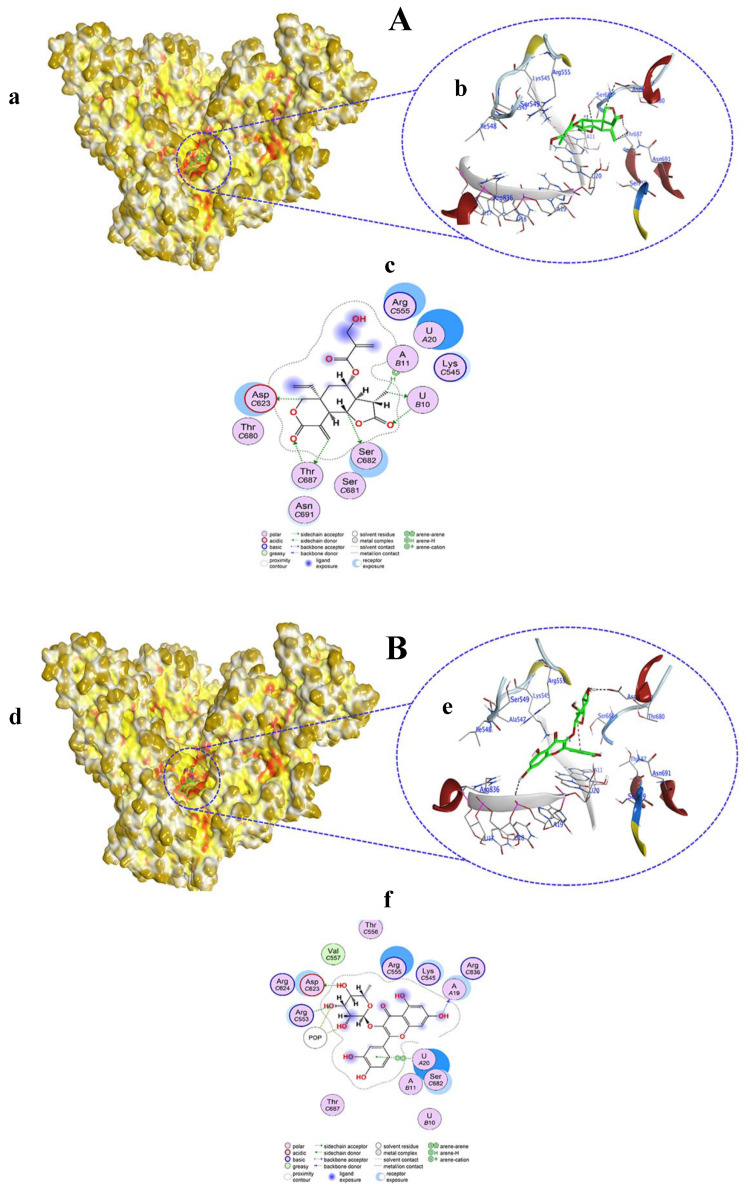
Surface representation showing the non-covalent docking of compound **9** (**A**(**a**)) and compound **10** (**B**(**d**)) with RdRp. Compounds **9** and **10** are in green color. Magnified view showing the binding of compound **9** (**A**(**b**)) and compound **10** (**B**(**e**)) with the template primer nucleotides and residues in the active pocket of RdRp. Two-dimensional view of RdRp showing non-covalent binding with compound **9** (**A**(**c**)) and compound **10** (**B**(**f**)).

Compound **9** predominantly acted as an H-donor and an H-acceptor within the active pocket of RdRp and interacted with both nucleotides and amino acid residues (Figure 7A(a,b)). In particular, C11, C15, and C21 interacted with SER682, ASP623, and THR687 residues at distances of 3.2, 3.3, and 3.36 Å, respectively. Conversely, the C19 (3.33 Å) atom of compound **9** interacted with the U10 nucleotide of RdRp through H-donor properties. On the other hand, as an H-acceptor, the O4 and O5 atoms of compound **9** showed interactions with a nucleotide (U10) and amino acid (THR687) at distances of 2.98 and 2.86 Å, respectively. H–π interaction was found between the C19 (4.61 Å) atom of compound **9** and the A11 nucleotide of RdRp (Table S10, Figure 7A(c)). 

Quercitrin 3-O-rhamnoside (compound **10**) docked in the active pocket of the RdRp protein, where it was bound with different amino acid residues and nucleotides by acting as an H–donor, H–acceptor, and H–π interaction (Figure 7B(d,e)). POP1003, POP1003, and ASP623 amino acids interacted with O43 (3.04 Å), O47 (3.23 Å), O51 (2.84 Å) atoms of compound **10,** acting as H-donors. Conversely, the O47 (3.17 Å) and O47 (2.99 Å) atoms of compound **10** interacted with the ARG553 residue via the H-acceptor process. O21 (2.92 Å) and six-ring (3.75 Å) atoms of compound **10** bound with A19 and U20 nucleotides of RdRp through H–acceptor and π–π interactions (Table S11, Figure 7B(f)). Compound **10** docking score was −8.1866 kcal/mol, which was near to the score of −8.870 obtained from the co-crystalized ligand remdesivir (RTP) with RdRp.

### 3.5. Irreversible (Covalent) Docking of Compounds ***1***–***10*** with RdRP

**Figure 8:**
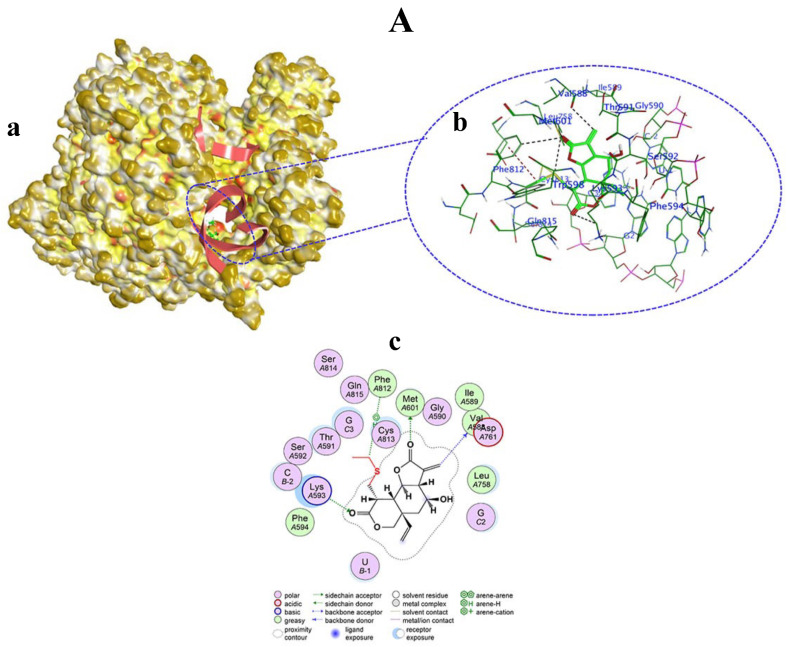
Covalent docking of Vernolepin (compound **7**) to SARS-CoV-2 target protein (RdRp) (**A**). (**a**) Surface representation of RdRp docked with compound **7** (green color). Solvent exposed region of RdRp is dark yellow, hydrophobic regions are yellow, and polar regions are red color. (**b**) Magnified view of the active pocket occupied by compound **7** showing interaction with residues in RdRp. H-bond (black color), H–π bond (dark red), Van der Waals clashes (dark blue), atoms (element color), and residues are labeled as blue texts. (**c**) Two-dimensional view of RdRp showing the interaction of amino acid residues with compound **7**. Descriptions of bonds and color of 2D are shown in the inset of (**c**).

Among the tested compounds, only vernolepin (compound **7**), vernadolol (compound **8**), and 11β,13-dihydrovernodalin (compound **9**) showed covalent bond formation with RdRp. Compound **7**, as a ligand, formed a covalent bond with its carbon atom in the vinyl group and a sulfur (SG) atom of CYS813 in RdRp (Figure 8A(a,b)). Apart from this, other interactions also developed between compound **7** and RdRp through hydrogen–donor, hydrogen–acceptor, and H–π processes. The O32 and C34 atoms of compound **7** developed a hydrogen bond by donating hydrogen between sulfur (SD) of MET601 and oxygen (O) of VAL588. The distance between O32 and SD was 3.47 Å (−0.5 kcal/mol), and the distance between C34 and O was 3.16 Å (−0.3 kcal/mol). Also, the O20 atom of compound **7** accepted hydrogen from the carbon (CE) atom of LYS593 at a distance of 3.27 Å (−0.3 kcal/mol). Moreover, the C5 atom of compound **7** formed a hydrogen bond by H–π interaction with the six ring of PHE812 residue at a distance of 4.58 Å and −0.4 kcal/mol energy (Table 1, Figure 8A(c)). 

**Figure 9:**
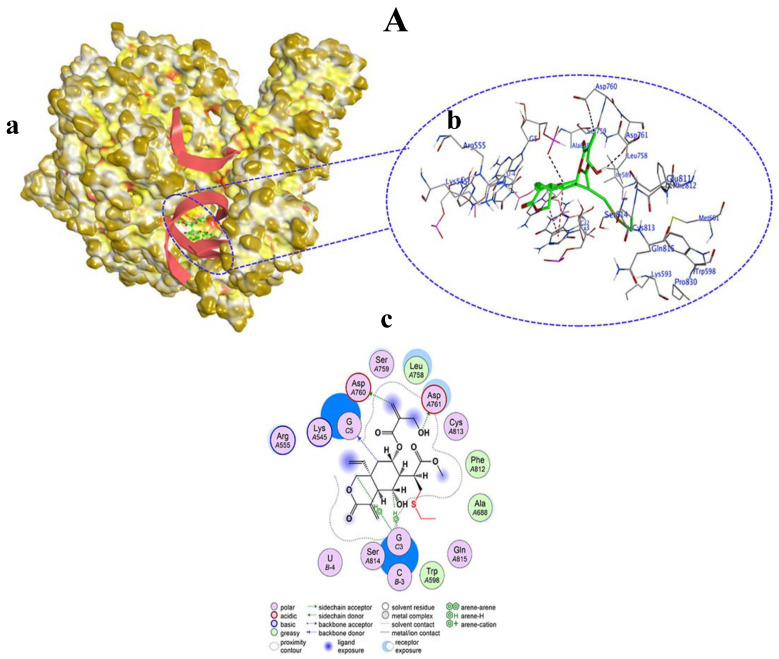
(**A**) Vernadolol (compound **8**) covalently bound to SARS-CoV-2 target protein (RdRp). (**a**) Surface representation of RdRp docked with compound **8** (green color). Solvent exposed region of RdRp is dark yellow, hydrophobic regions are yellow, and polar regions are red in color. (**b**) Magnified view of the active pocket occupied by compound **8** showing interaction with residues in RdRp. H-bond (black color), H–π bond (dark red), Van der Waals clashes (dark blue), atoms (element color), and residues are labeled as blue texts. (**c**) Two-dimensional view of RdRp showing the interaction of amino acid residues with compound **8**. Descriptions of bonds and color of 2D are shown in the inset of (**c**).

Vernadolol (compound **8**) also developed a covalent bond with the carbon atom of the vinyl group and the sulfur atom (SG) of CYS813 in RdRp. In addition, hydrogen bonds also developed between the ligand (compound **8**) and the receptor (RdRp) atoms (Figure 9A(a,b)). Compound **8** donated hydrogen to oxygen (OD2) of ASP76 and ASP761 amino acid residues at distances of 3.59 Å and 3.32 Å with −0.34 and −1.7 kcal/mol energy, respectively. The C59 atom of compound **8** donated hydrogen to the oxygen atom (OP2) of guanine (G5) in the template primer. Also, the C15 and C27 atoms of compound **8** developed hydrogen bonds with the five ring of guanine (G3) at distances of 3.63 and 4.37 Å, respectively. Overall, the interaction between compound **8** and RdRp exhibited a high docking score of −13.482 kcal/mol (Table 2, Figure 9A(c)). 

**Figure 10:**
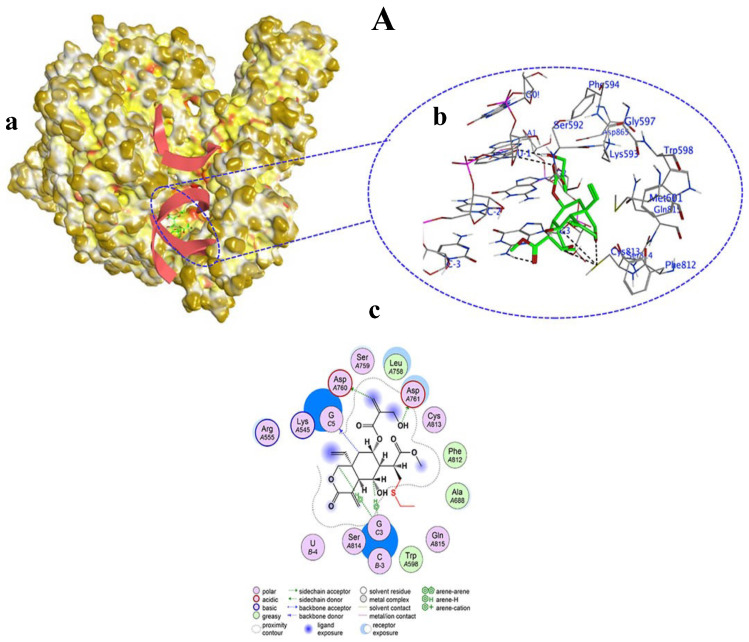
(**A**) 11β,13-Dihydrovernodalin (compound **9**) covalently bound to SARS-CoV-2 target protein (RdRp). (**a**) Surface representation of RdRp docked with compound **9** (green color). Solvent exposed region of RdRp is dark yellow, hydrophobic regions are yellow, and polar regions are red in color. (**b**) Magnified view of the active pocket occupied by compound **9** showing interaction with residues in RdRp. H-bond (black color), H–π bond (dark red), Van der Waals clashes (dark blue), atoms (element color), and residues are labeled as blue texts. (**c**) Two-dimensional view of RdRp showing the interaction of amino acid residues with compound **9**. Descriptions of bonds and color in 2D are shown in the inset of (**c**).

11β,13-Dihydrovernodalin’s (compound **9**) interaction with the target protein (RdRp) also resulted in covalent bond formation with its carbon atom in the vinyl group and a sulfur atom (SG) in the CYS813 amino acid. Apart from covalent bond formation, several non-covalent interactions also took place, which led to the formation of hydrogen bonds (Figure 10A(a,b)). Specifically, oxygen (O15) of compound **9** interacted with the OD2 of ASP761 to donate hydrogen and developed a hydrogen bond at a distance of 3 Å showing −1.1 kcal/mol energy for this binding. A hydrogen bond also developed between the carbon atom (C29) of compound **9** and the O2 of uracil (U) in the RdRP template primer at a distance of 3.12 Å. A H–π interaction developed between C18, C19, and C27 atoms of compound **9** and the guanine (G) nucleotides of RdRp. The overall docking score for the interaction between compound **9** and RdRP was −6.732 kcal/mol (Table 3, Figure 10A(c)). 

### 3.6. Stability, Conformity, and Compactness Analysis by MD Simulation 

#### 3.6.1. RMSD Measurements

**Figure 11:**
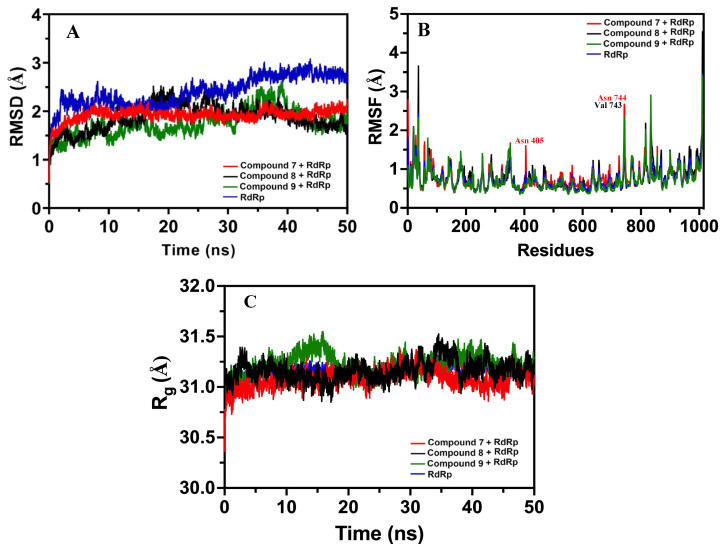
MD simulation analysis of RdRp and its complexation with compounds **7**, **8**, and **9**. (**A**) RMSD of RdRp native structure and post-complexation with compounds **7**, **8**, and **9** at 50 ns. (**B**) RMSF of RdRp alone and after their complexation with compounds **7**, **8**, and **9**. (**C**) Rg of RdRP showing the amino acid compactness in the absence and presence of compounds **7**, **8**, and **9** as a function of time.

The RMSD of backbone RdRp was compared with the RMSD of RdRp separately complexed with compounds **7**, **8**, and **9**. RMSD representing the fluctuations in the average position of ligands (compounds **7**, **8**, and **9**) and RdRp is shown in Figure 11A. The RMSD values exhibited the average deviation of compounds **7**, **8**, and **9** positions from their initial reference structure during 50 ns. RMSD data exhibited that compounds **7**, **8**, and **9** formed relatively stable interactions with RdRp. Compounds **7**, **8**, and **9** displayed average RMSD values of 1.922 ± 0.122, 1.867 ± 0.267 Å, and 1.757 ± 0.284 Å, respectively. Overall, the RMSD and SD values of compounds **7** and **9** were nearby each other and lower than the RMSD value of 2.403 ± 0.309 Å RdRp alone, indicating decreased fluctuation and more stable complex formation as compared to the target alone. 

#### 3.6.2. Quantification of RMSF and Rg

The RMSF value of RdRp backbone Cα atoms was calculated by averaging all conformations during the 50ns simulation time. The apo-RdRP showed large fluctuations of residues in the interface, finger, and loop region. Also, residues in the proximity of the N- and C-terminal showed greater fluctuation. Relatively so, the majority of RdRp residues when bound to compounds **7**, **8**, and **9** showed no significant changes (>0.5 Å) in the fluctuation of residues. Compound **7** caused significant fluctuations of 0.67 Å and 0.56 Å only in ASN405 and ASN744 residues, respectively. Compound **8** binding showed a significant fluctuation of only VAL743 (0.56 Å). Conversely, compound **9** binding did not cause any significant change (Figure 11B). RdRp in the absence of ligands showed Rg of 31.15 ± 0.058 Å, which was changed to 31.07 ± 0.086 Å, 31.16 ± 0.97 Å, and 31.21 ± 0.101 Å after complexation with compounds **7**, **8**, and **9**, respectively (Figure 11C).

## 4. Discussion

As soon as information on the structure of SARS-CoV-2 came into the public domain, the search for potent synthetic drugs as antiviral inhibitors accelerated [2,16,20,61,62,63,64]. After a couple of years of the pandemic, several countries faced second or third waves of COVID-19 caused by the mutant variants of SARS-CoV-2 [65]. Fortunately, to protect humans against SARS-CoV-2 infection, a few vaccines and monoclonal antibodies are available [66,67]. Recently, small-molecule drugs (remdesivir, molnupiravir, and nirmatrelvir) have been introduced as treatment options that act as nucleoside analogs to inhibit viral replication and covalently bind to inhibit viral essential protease [68,69,70]. In spite of this fact, such therapeutic advancements do have some limitations. For example, vaccines, during the course of time, lose their efficacy and need periodic booster shots. They also need the development of new vaccines against variants [71]. Distantly, the production of monoclonal antibodies is a costly affair and prone to viral evasion, owing to mutation [66,72]. In its latest announcement, the USFDA has approved the use of paxlovid (nirmatrelvir and ritonavir tablets) as the first oral antiviral for adults infected with COVID-19 [73]. Also, veklury (remdesivir) is another choice, but it must be given by intravenous infusion, and it is more effective than molnupiravir, which is given orally; however, they are used for the targeted control of mild to moderate symptoms of COVID-19 infection [74,75]. Apart from the encouraging effects of novel therapeutic innovations, there is still urgency to discover highly selective antiviral agents that possess the specificity to inhibit SARS-CoV-2 multiplication. In particular, efforts are being made by researchers and scientists to look for novel antiviral compounds from natural sources that have the ability to inhibit viral multiplication to prevent its progression towards a deadly condition. For decades, humans have used natural flora for therapeutic purposes. As a result, bioactive phytochemicals have been identified that possess medicinal values and are used for healthy living and to cure diseases. The medicinal properties of plant extracts chiefly depend on the presence of a variety of active ingredients, mineral deposits, vitamin supplements, and secondary metabolites that have fewer side effects [37]. There are some recent pieces of evidence that indicates that phytochemicals have the ability to interact with non-structural proteins (3CL^Pro^, RdRp, PL^Pro^) of SARS-CoV-2, indicating their suitability as prospective drugs [13,31,76,77,78,79,80]. 

RdRp’s core protein is a single chain consisting of approximately 900 amino acid residues. The enhanced activity of the SARS-CoV-2 core protein relies on the association of nps7/8, as well as an additional nsp8 protein [81]. RdRp shows a “finger–palm–thumb” conformation due to several conserved motifs, and it uses two sequential aspartate residues coordinated by Mg^++^ ions as their catalytic centers [82]. The C-terminal polymerase domain of RdRp has PHE920 and SER367 residues, which play an essential role in the transcription and replication of virus [18,83]. In motif C, ASP760, ASP761, and SER759 are catalytic residues that are required for RNA synthesis. On the other hand, in motif A, the ASP618 residue is liable for divalent cation binding [83]. RdRP is highly conserved, and its homologue is absent in the host. Consequently, it is a hot spot target for the potential antiviral drug [82]. In this regard, we relied on the pharmacological properties of those ten compounds (compounds **1**–**10**) that our lab has previously tested in different assays [40,41,42]. Hence, we implemented a computational approach to evaluate the efficacy of compounds **1**–**10** to inhibit RdRp, a crucial replicative enzyme vital for its multiplication. To that end, first, we confirmed the protocol implemented for the docking of remdesivir (RTP) and RdRp so that the prospective data obtained from it must display conformity, stability, and specificity. We found that RTP- RdRp interaction clearly revealed the usefulness and consistency of the docking protocol that we have used in our study. Thereupon, a reversible (non-covalent) docking protocol was implemented to classify specific inhibitors among the cohort of compounds **1**–**10**. Later, we implemented the irreversible (covalent) docking method to find those compounds that exhibit strong bonding within the active site of RdRp. 

The non-covalent docking data of compounds **1**–**10** with RdRP exhibited their preferential interactions with template primer nucleotides (U and A). However, the interactions of compounds **1**–**10** with amino acid residues were very selective. In particular, we found that compounds **1**, **2**, **3**, **4**, **5**, **6**, **8**, **9**, and **10** formed hydrogen bonds with key amino acid residues (ASP623, LYS545, ARG555, ASN691, SER682, and ARG553) in the active site of RdRp. The selective interaction of the above compounds imitated the interaction behavior of remdesivir, which showed binding with the uridine (U) base and side chain residues (LYS545 and ARG555), which are essential for the catalytic activity of RdRp [84,85,86]. ASP623, SER682, and ASN691 residues in RdRp govern the role of 2′-OH recognition of incoming nucleotides [87]. In fact, the above-listed compounds shared the same residues for interaction with RdRp, as also reported for US FDA-approved drugs (carbetocin, examorelin, lanreotide, lypressin) and plant-derived chemicals [81,88]. On the other hand, when comparing the docking score criteria, it was noticeable that compound **2** (−7.736 kcal/mol), **3** (−8.026 kcal/mol), and **10** (−8.186 kcal/mol) values were close to the docking score of the antiviral drug remdesivir (−8.2 kcal/mol) [89]. The encouraging non-covalent data motivated us to evaluate the covalent binding potential of compounds **1**–**10** with RdRp. In this line, we used the MOE covalent docking module, which primarily relies on the reaction placement methodology to match the reactive group on ligand and cysteine residue, leading to the formation of a covalent bond between them. However, other important tools like AutoDock4, CovDock, FITTED, GOLD, and ICM-Pro also emphasize scenarios where these programs may offer benefits or outperform MOE, owing to their unique scoring functions or algorithms [90]. In covalent docking, we found only three compounds (i.e., compounds **7**, **8**, and **9**) exhibited their irreversible (covalent bond) nature of interaction with CYS813, a crucial amino acid in the palm domain of RdRP. Covalent inhibitors offer manifold advantages including low-dose administration, higher potency, prolonged inhibition time, being less prone to pharmacokinetic parameters, higher selectivity and biochemical efficiency, and low risk of drug resistance. Few disadvantages of covalent inhibitor embody the unexpected hypersensitivity or toxicity, putative immunogenicity of target adducts, requirement of activated and accessible nucleophiles, and seemingly not suitable nature for targets that have fast enzyme turnover [91]. It is vital to recognize the benefits and drawbacks can be affected by the specific biological target and the molecular design of the covalent inhibitor, highlighting the prerequisite for careful optimization in the drug development process. 

Relatively, the advantageous aspects of non-covalent inhibitor drugs exemplify the fact that it is significantly easier to evade toxicity and the non-requirement of strong nucleophiles, and a large library of such drugs is available. This is notwithstanding the fact that non-covalent inhibitors possess low selectivity, are less potent, are restricted to non-covalent binding affinity, and have exhibited mostly poor reactivity [91]. In this line, compounds **7**, **8**, and **9** also showed a reversible (non-covalent) interaction with other amino acids (LYS593, VAL588, MET601, ASP760, and ASP761) in the palm domain of RdRp. SARS-CoV-2 RdRp, or nsp12, comprises the C-terminal polymerase domain, which encompasses amino acid residues from SER367 to PHE920 and plays a vital role in the replication and transcription of a virus [18,83,92]. Within this domain, a few conserved amino acid residues (ASP760, ASP761, and SER759) as well as other crucial residues (CYS813, ARG555, SER549, THR190, LYS551, GLN815, and SER814) are hot spots for covalent interaction with inhibitors [59]. Notwithstanding this fact, CYS813 and ASP761 are crucial for the interaction with the nsp7/8 complex [93]. We have also found that compounds **8** and **9** have interaction capabilities with the nucleotides in the template primer of RdRp. In the same line, the antiviral drug remdesivir also showed its interaction with the uridine bases in the template strand to inhibit the function of the replication process of RdRp [86]. It is intriguing to find that the covalent docking scores of compounds **7** and **9** (−6.136 and −6.732 kcal/mol, respectively) fell near the docking score obtained with remdesivir (−8.2 kcal/mol), while compound **8** exhibited a much higher negative docking score (−13.482 kcal/mol), affirming its stronger association with this target. Our data corroborate with some in silico findings on phytochemicals, demonstrating their inhibitory effects on the replicative potential of RdRp [18,37,38,89]. Correlative to the non-covalent docking approach, the covalent docking method we used is new and tough to implement, albeit, covalent docking currently captures sizeable attention over non-covalent docking. Covalent docking delivers both superior efficacy and potency, greater stability, and a prolonged period of action with the target through the irreversible nature of action to repress resistance [94,95,96].

Viewing the promising effects of compounds **7**, **8**, and **9**, we further analyzed their influences on the structure and conformation of RdRp to help understand their putative inhibition effects on the target. We performed the MD simulation analysis by first measuring the RMSD to unravel the stability of compounds **7**, **8**, and **9** with RdRp. The stimulated structure of RdRP in the absence and presence of the above compounds demonstrated very little variation after their complexation with RdRP, as also evidenced by the RMSD data, which were either lessened or close to the value of the native structure of RdRp. Such a response implies the reliability of compounds **7**, **8**, and **9** that they were not prone to divert and were explicitly stable within the active pocket of RdRp. The stable responses of our compounds to RdRp are in agreement with similar responses of different phytochemicals in general and traditional Chinese herbs against RdRp [18,37]. Subsequently, we performed RMSF analysis to evaluate the structural fluctuations as well as flexibility of per residue in RdRp. Only two residues, ASN405 in the finger domain and ASN744 in the palm domain, showed marginal but significant fluctuation by compound **7**. On the other hand, only one residue (VAL743 in the palm domain) exhibited significant fluctuation by compound **8**. Conversely, compound **9** did not show significant fluctuations. Following the threshold criteria of significant change of fluctuations >5 Å [97,98], we found that the rest of the residues in RdRP showed no significant fluctuations upon their complexation with compounds **7**, **8**, and **9**, affirming their stability during the simulation period [99,100]. In the same line, Rg was evaluated, which enabled us to measure changes in the compactness as well as the stable folding or unfolding of the ligand–protein complex during the simulation period. Higher Rg indicated a lower compactness of the ligand–protein complex [100]. We found that the native RdRp as well as when it was complexed with compounds **7**, **8**, and **9** exhibited almost similar average Rg values. Such behavior indicated that if a protein maintains the steady value of Rg during the whole period of MD simulation, it can be regarded as the protein is stably folded; if Rg changes with time, then the protein is unfolded [100].

## 5. Conclusions

We computationally studied the effectiveness of ten phytochemicals (compounds **1**–**10**) to bind with RdRp, which is essentially required for the replication of SARS-CoV-2. We implemented the non-covalent (reversible) docking protocol and found that compounds **1**–**10** possess the capability to bind with different amino acid residues as well as the template primer nucleotides. The covalent (irreversible) docking compounds **7**–**9** showed their capabilities to form covalent bonds with CYS813 in the palm domain. Subsequently, MD simulation analysis by RMSD, RMSF, and Rg analyses affirmed the stability of compounds in the active pocket of RdRP. The tested compounds unequivocally demonstrated in silico inhibitory potential towards RdRP; their efficacy as anti-SARS-CoV-2 agents warrants further studies using suitable in vitro and in vivo test models. 

## Figures and Tables

**Table 1:** Covalent docking protocol resulting in auxiliary interactions (hydrogen bond formation) by vernolepin (compound **7**) with amino acid residues in the active pocket of the SARS-CoV-2 target protein (RdRp).

Ligand (Compound 7)	Receptor (RdRp)	Interaction Type	Distance (Å)	Energy (kcal/mol)	Docking Score (kcal/mol)
O 32	SD MET 601 (A)	H–donor	3.47	−0.5	−6.136
C 34	O VAL 588 (A)	H–donor	3.16	−0.3	
O 20	CE LYS 593 (A)	H–acceptor	3.27	−0.3	
C 5	Six-ring PHE 812 (A)	H–π	4.58	−0.4	

**Table 2:** Covalent docking protocol resulting auxiliary interactions (hydrogen bond formation) by vernadolol (compound **8**) with amino acid residues in the active pocket of SARS-CoV-2 target protein (RdRp).

Ligand (Compound 8)	Receptor (RdRp)	Interaction Type	Distance (Å)	Energy (kcal/mol)	Docking Score (kcal/mol)
C 51	OD2 ASP 760 (A)	H–donor	3.59	−0.3	−13.482
O 57	OD2 ASP 761 (A)	H–donor	3.32	−1.7	
C 59	OP2 G 5 (G)	H–donor	3.28	−0.6	
C 15	Five-ring G 3 (G)	H–π	3.63	−0.3	
C 27	Five-ring G 3 (G)	H–π	4.37	−0.3	

**Table 3:** Covalent docking protocol resulting in auxiliary interactions (hydrogen bond formation) by 11β,13-dihydrovernodalin (compound **9**) with amino acid residues in the active pocket of the SARS-CoV-2 target protein (RdRp).

Ligand (Compound 9)	Receptor (RdRp)	Interaction Type	Distance (Å)	Energy (kcal/mol)	Docking Score (kcal/mol)
O 15	OD2 ASP 761 (A)	H–donor	3	−1.1	−6.732
C 29	O2 U −4 (F)	H–donor	3.12	−0.3	
C 18	Six-ring G 3 (G)	H–π	4.42	−0.3	
C 19	Five-ring G 5 (G)	H–π	3.68	−0.3	
C 27	Five-ring G 3 (G)	H–π	4.35	−0.5	

## Data Availability

All data are provided in this manuscript.

## Supplementary Materials

The following supporting information can be downloaded at: https://www.mdpi.com/article/10.3390/v15112175/s1, Figure S1: Validation of docking protocol with RdRp and co-crystal ligand remdesivir (RTP). Table S1: Validation of redocking interaction between co-crystal ligand remdesivir (RTP) with SARS-CoV-2 target protein (RdRp). Tables S2–S11: Non-covalent docking of 5,3′,4′-trihydroxyflavan 7-O-gallate (compound **1**–**10**) with SARS-CoV-2 target protein (RdRp).

## Abbreviations

RNA-dependent RNA polymerase (RdRp), aspartic acid (ASP), lysine (LYS), arginine (ARG), asparagine (ASN), serine (SER), cysteine (CYS), aspartic acid (ASP), molecular dynamic simulation (MD), threonine (THR), methionine (MET), valine (VAL), phenylalanine (PHE), glutamine (GLN), pyrophosphate (POP), adenine (A), uracil (U), guanine (G), root-mean-square deviation (RMSD), root-mean-square fluctuation (RMSF), radius of gyration (Rg), severe acute respiratory syndrome coronavirus 2 (SARS-CoV-2), coronavirus disease 2019 (COVID-19), acute respiratory distress syndrome (ARDS), angiotensin-converting enzyme 2 (ACE2), non-structural proteins (NSPs), main protease (M^Pro^), papain-like protease (PL^Pro^), non-structural proteins 7 and 8 (NSP7/8), 5,3′,4′-trihydroxyflavan 7-O-gallate (compound **1**), 5,4′-dihydroxyflavan 7-3′-O-digallate (compound **2**), 5,3′-dihydroxyflavan 7-4′-O-digallate (compound **3**), spinasterol (compound **4**), stigmasterol (compound **5**), 3′,4′,5,7-tetrahydroxy-3-methoxyflavone (compound **6**), vernolepin (compound **7**), vernadolol (compound **8**), and 11β,13-dihydrovernodalin (compound **9**), quercitrin 3-O-rhamnoside (compound **10**), remdesivir (RTP), Protein Data Bank (PDB), Molecular Operating Environment (MOE), Nanoscale molecular dynamics (NAMD), CHARMM General Force Field (CGenFF), transferable intermolecular potential 3P (TIP3P), constant-volume ensemble (NVT), constant-pressure ensemble (NPT).
