# Supplementary material for: Covalent Inhibitors from Saudi Medicinal Plants Target RNA-Dependent RNA Polymerase (RdRp) of SARS-CoV-2
Source: Viruses. 2023 Oct 30;15(11):2175. doi: 10.3390/v15112175 (PMC10675690; doi:10.3390/v15112175)
Supplement: Supplementary file 1 [file viruses-15-02175-s001.zip › Supplementary Figures and Tables.pdf]

Supplementary Table S1. Validation of redocking interaction between co-crystal ligand Remdesivir (RTP) with SARS-CoV-2 target protein (RdRp).

Ligand (RTP)	Receptor (RdRp)	Interaction Type	Distance (Å)	Energy (kcal/mol)	Docking Score (kcal/mol)
N5 29	O4 U 10 (T)	H-donor	2.98	-3.2	-8.870
N4 16	N3 U 10 (T)	H-acceptor	3.06	-5.6	
O6 19	O3' U 20 (P)	H-acceptor	2.91	-2.9	
O1 32	O3' U 20 (P)	H-acceptor	3.4	-0.5	
O5 35	MG MG 1004 (A)	Metal	1.93	-5.2	
O5 35	MG MG 1004 (A)	Ionic	1.93	-17.5	
5-ring	6-ring U 20 (P)	π-π	3.89	0	
6-ring	6-ring A 11 (T)	π-π	3.87	0	

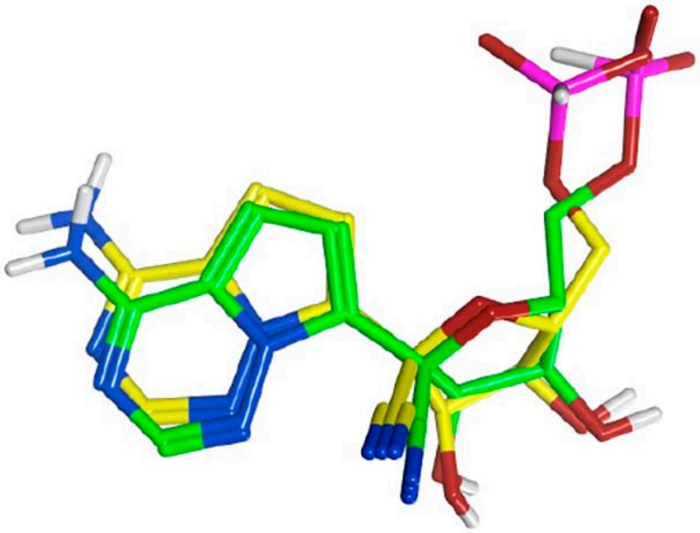

Supplementary Figure S1. Validation of docking protocol with RdRp and co-crystal ligand Remdesivir (RTP). Superimposed image after redocking of co-crystallized ligand Remdesivir (RTP) (green) with the crystalized ligand (yellow) RSMD: 0.970 Å.

Supplementary Table S2. Non-covalent docking of 5,3′,4′-trihydroxyflavan 7-O-gallate (compound **1**) with SARS-CoV-2 target protein (RdRp).

Ligand (Compound 1)	Receptor (RdRp)	Interaction Type	Distance (Å)	Energy (kcal/mol)	Docking Score (kcal/mol)
O 1	N7 A 19 (P)	H-donor	2.95	-3.6	-6.8
O 5	OP2 U 18 (P)	H-donor	2.93	-0.6	
O 31	OD1 ASP 623 (A)	H-donor	3.18	-0.6	
O 37	O2 U 20 (P)	H-donor	3.09	-0.9	
O 41	NZ LYS 545 (A)	H-acceptor	3.13	-0.8	
C 39	6-ring U 20 (P)	H-π	4.17	-0.7	

Supplementary Table S3. Non-covalent docking of 5,4′-dihydroxyflavan 7-3′-O-digallate (compound **2**) with SARS-CoV-2 target protein (RdRp).

Ligand (Compound 2)	Receptor (RdRp)	Interaction Type	Distance (Å)	Energy (kcal/mol)	Docking Score (kcal/mol)
O 13	OP2 U 10 (T)	H-donor	2.87	-4.6	-7.7
O 42	O5 POP 1003 (A)	H-donor	2.78	-5.6	
C 62	6-ring U 20 (P)	H-π	4.48	-0.2	
6-ring	CD LYS 545 (A)	π-H	4.54	-0.5	
6-ring	CD ARG 555 (A)	π-H	4.13	-0.2	

Supplementary Table S4. Non-covalent docking of 5,3′-dihydroxyflavan 7-4′-O-digallate (compound **3**) with SARS-CoV-2 target protein (RdRp).

Ligand (Compound 3)	Receptor (RdRp)	Interaction Type	Distance (Å)	Energy (kcal/mol)	Docking Score (kcal/mol)
O 10	OP2 U 18 (P)	H-donor	2.81	-3.6	-8.0267
O 13	OP2 U 17 (P)	H-donor	3	-3.7	
O 60	O5 POP 1003 (A)	H-donor	2.91	-2	
C 30	6-ring U 20 (P)	H-π	4.43	-0.2	
6-ring	NZ LYS 545 (A)	π-cation	4.28	-0.2	

Supplementary Table S5. Non-covalent docking of Spinasterol (compound **4**) with SARS-CoV-2 target protein (RdRp).

**Ligand (Compound 4)**	**Receptor (RdRp)**	**Interaction Type**	**Distance (Å)**	**Energy (kcal/mol)**	**Docking Score (kcal/mol)**
O 1	OG1 THR 680 (A)	H-donor	3.1	-0.2	-5.0536
O 1	ND2 ASN 691 (A)	H-acceptor	2.94	-2	

Supplementary Table S6. Non-covalent docking of Spinasterol (compound **5**) with SARS-CoV-2 target protein (RdRp).

**Ligand (Compound 5)**	**Receptor (RdRp)**	**Interaction Type**	**Distance (Å)**	**Energy (kcal/mol)**	**Docking Score (kcal/mol)**
O 1	OG1 THR 680 (A)	H-donor	3.1	-0.2	-4.7243
O 1	ND2 ASN 691 (A)	H-acceptor	2.94	-2	

Supplementary Table S7. Non-covalent docking of 3′,4′,5,7-tetrahydroxy-3-methoxyflavone (compound **6**) with SARS-CoV-2 target protein (RdRp).

**Ligand (Compound 6)**	**Receptor (RdRp)**	**Interaction Type**	**Distance (Å)**	**Energy (kcal/mol)**	**Docking Score (kcal/mol)**
O 19	O5 POP 1003 (A)	H-donor	3.17	-3	-6.8664
O 1	N3 U 10 (T)	H-acceptor	3.27	-1.1	
O 1	NZ LYS 545 (A)	H-acceptor	3.49	-1.1	
O 27	N3 U 10 (T)	H-acceptor	3.3	-0.7	
C 11	6-ring U 20 (P)	H-π	4.51	-0.3	
6-ring	C2' U 20 (P)	π-H	4.98	-0.3	

Supplementary Table S8. Non-covalent docking of Vernolepin (compound **7**) with SARS-CoV-2 target protein (RdRp).

**Ligand (Compound 7)**	**Receptor (RdRp)**	**Interaction Type**	**Distance (Å)**	**Energy (kcal/mol)**	**Docking Score (kcal/mol)**
O 32	O5 POP 1003 (A)	H-donor	2.85	-3.5	-5.9227
O 12	N3 U 10 (T)	H-acceptor	2.99	-4.3	
C 14	6-ring A 11 (T)	H-pi	4.88	-0.2	

Supplementary Table S9. Non-covalent docking of Vernadolol (compound **8**) with SARS-CoV-2 target protein (RdRp).

Ligand (Compound 8)	Receptor (RdRp)	Interaction Type	Distance (Å)	Energy (kcal/mol)	Docking Score (kcal/mol)
O 12	SG CYS 622 (A)	H-donor	3.55	-0.2	-6.4809
O 21	O5 POP 1003 (A)	H-donor	2.86	-3.5	
C 42	O2 U 10 (T)	H-donor	3.43	-0.4	
O 30	NZ LYS 545 (A)	H-acceptor	3.2	-0.2	
O 31	NZ LYS 545 (A)	H-acceptor	3.28	-1.3	
O 48	NZ LYS 545 (A)	H-acceptor	2.87	-2.5	
O 48	6-ring U 20 (P)	H-pi	4.62	-0.9	

Supplementary Table S10. Non-covalent docking of 11β,13-dihydrovernodalin (compound **9**) with SARS-CoV-2 target protein (RdRp).

Ligand (Compound 9)	Receptor (RdRp)	Interaction Type	Distance (Å)	Energy (kcal/mol)	Docking Score (kcal/mol)
C 11	OG SER 682 (A)	H-donor	3.2	-0.2	-6.4809
C 15	OD1 ASP 623 (A)	H-donor	3.3	-0.5	
C 19	O4 U 10 (T)	H-donor	3.33	-0.3	
C 21	OG1 THR 687 (A)	H-donor	3.36	-0.2	
O 4	N3 U 10 (T)	H-acceptor	2.98	-4.1	
O 5	OG1 THR 687 (A)	H-acceptor	2.86	-0.6	
C 19	6-ring A 11 (T)	H-pi	4.61	-0.3	

Supplementary Table S11. Non-covalent docking of Quercitrin 3-O-rhamnoside (compound **10**) with SARS-CoV-2 target protein (RdRp).

Ligand (Compound 10)	Receptor (RdRp)	Interaction Type	Distance (Å)	Energy (kcal/mol)	Docking Score (kcal/mol)
O 21	OP2 A 19 (P)	H-donor	2.92	-3.7	-8.1866
O 43	O5 POP 1003 (A)	H-donor	3.04	-1.6	
O 47	O5 POP 1003 (A)	H-donor	3.23	-1.6	
O 51	OD1 ASP 623 (A)	H-donor	2.84	-4.7	
O 47	NH1 ARG 553 (A)	H-acceptor	3.17	-0.4	
O 47	NH2 ARG 553 (A)	H-acceptor	2.99	-1.4	
6-ring	6-ring U 20 (P)	pi-pi	3.75	-0.1	
